# Nanocarbon Black and Molybdenum Disulfide Hybrid Filler System for the Enhancement of Fracture Toughness and Electromechanical Sensing Properties in the Silicone Rubber-Based Energy Harvester

**DOI:** 10.3390/polym15092189

**Published:** 2023-05-05

**Authors:** Md Najib Alam, Vineet Kumar, Taemin Jeong, Sang-Shin Park

**Affiliations:** School of Mechanical Engineering, Yeungnam University, 280, Daehak-ro, Gyeongsan 38541, Republic of Korea; mdnajib.alam3@gmail.com (M.N.A.); vineetfri@gmail.com (V.K.); jjtt00mm@naver.com (T.J.)

**Keywords:** silicone rubber, nanocarbon black, molybdenum disulfide, nanocomposites, energy harvester

## Abstract

Recently, hybrid fillers have been found to be more advantageous in energy-harvesting composites. This study investigated the mechanical and electromechanical performances of silicone rubber-based composites made from hybrid fillers containing conductive nanocarbon black (NCB) and molybdenum disulfide (MoS_2_). A hybrid filler system containing only 3 phr (per hundred grams of rubber) MoS_2_ and 17 phr NCB provided higher fracture strain, better tensile strength, and excellent toughness values compared to the 20 phr NCB-only-filled and 5 phr MoS_2_-only-filled rubber composites. The chemical cross-link densities suggest that NCB promoted the formation of cross-links, whereas MoS_2_ slightly reduced the cross-link density. The higher mechanical properties in the hybrid filler systems suggest that the filler particles were more uniformly distributed, which was confirmed by the scanning electron microscope study. Uniformly distributed filler particles with moderate cross-link density in hybrid filler systems greatly improved the fracture strain and fracture toughness. For example, the hybrid filler with a 17:3 ratio of NCB to MoS_2_ showed a 184% increment in fracture toughness, and a 93% increment in fracture strain, compared to the 20 phr NCB-only-filled composite. Regarding electromechanical sensing with 2 kPa of applied cyclic pressure, the hybrid filler (17:3 CB to MoS_2_) performed significantly better (~100%) than the 20 phr NCB-only compound. This may have been due to the excellent distribution of conducting NCB networks and piezoelectric MoS_2_ that caused symmetric charging–discharging in the toughened hybrid composite. Thus, hybrid composites with excellent fatigue resistance can find dynamic applications, such as in blood pressure measurement.

## 1. Introduction

Recently, silicone rubber has become increasingly popular in various electronic, medical, and soft robotic applications, either directly or in combination with reinforcing fillers [1,2,3,4,5,6]. Silicone rubber is a highly dielectric material, and cannot transmit electricity. It exhibits a very high actuation value in the presence of electrical fields, considering its application in the field of soft robotics [7]. Inversely, it exhibits fluctuations in the electrical dipoles upon mechanical deformation, and converts mechanical energy to electrical energy. These properties make it valuable for applications in electromechanical pressure, strain, and other sensors [8,9].

To fabricate a soft and flexible electromechanical sensor, silicone rubber must be compounded with a conducting filler. Among various nanofillers, carbon black (CB), nano graphite (NG), and carbon nanotubes (CNTs) are the most suitable that can sufficiently improve the mechanical and electrical properties of silicone rubber [6]. CB is of particular interest because of its isotropic filler structure, excellent reinforcing properties, and good electrical properties. Owing to their isotropic shape, the composites are very stable, with fatigue in all directions; they are suitable for dynamic applications such as tires. Thus, electrodes made of CB in silicone rubber should maintain a uniform conductivity, which is necessary for electromechanical sensing.

Recently, instead of using a single filler, a hybrid filler was used to make rubber composites to reduce filler percolation and achieve synergistic mechanical and electrical properties [10]. The hybridization of different-dimensional fillers in rubber could be useful for improving filler dispersion, reducing filler percolation, and improving the stretchability of hybrid filler networks. These advantages make hybrid-filler-based rubber composites tougher than single-filler-based rubber composites. Furthermore, CB has recently been hybridized with silica [11], nano clay [12], graphene [13], and carbon nanotubes [14,15] in different polymers.

After the significant achievements of two-dimensional (2D) graphene in science and technology, many other 2D nanomaterials, such as hexagonal boron nitride, graphitic carbon nitride, and transition metal dichalcogenides (TMDs), have been investigated as alternatives to graphene [16,17]. Among the different TMDs, MoS_2_ has gained significant interest because of its electronic properties that are complementary to those of graphene. Unlike graphene, MoS_2_ has an intrinsic bandgap that enables its use in semiconducting electronic and optoelectronic applications [18,19]. Single-layered MoS_2_ has an extraordinary fracture strength of ~23 GPa and an elastic modulus of 300 GPa, which are similar to those of chemically reduced graphene [20]. Moreover, MoS_2_ is a piezoelectric material [21]. Therefore, MoS_2_ can be used as a reinforcing filler. Different polymer composites [22,23,24,25,26], including rubber composites [27,28,29,30,31], have been fabricated using modified and unmodified MoS_2_ particles for mechanical, microwave absorption, and tribology applications. Tang et al. [31] hybridized CB and 2D-MoS_2_ particles to reinforce natural rubber, and found about a 50% enhancement in the tensile modulus from a single CB-loaded composite. Although 2D MoS_2_ remarkably improves the properties of rubber composites, few studies have investigated silicone rubber composites for improving its toughness and its applications in electromechanical energy harvesting.

Reinforcing filler, with a high aspect ratio such as CNT, can simultaneously improve the electrical and mechanical moduli, but can significantly reduce the fracture strain [32]. Hence, a low aspect ratio with an electrically conductive filler could be useful to make a soft and stretchable energy harvester for low-energy sensing applications. This research aimed to improve the mechanical properties, especially the fracture strain and fracture toughness, of silicone rubber composites that can find suitable applications in energy harvesting devices for low mechanical energy sensitivity. Nano carbon black (NCB) is a very useful reinforcing filler for all types of rubber, owing to its considerable mechanical and electrical properties. NCB provides good electrical conductivity at 15–20 phr of filler amounts in silicone rubber [33]. In rubber-based flexible piezoresistive strain sensors, the sensitivity depends highly on the gauge factor. At low deformation, the gauge factor is significantly low. However, at higher deformation, the gauge factor could be enhanced, but the stability of the piezoresistive strain sensor gradually decreases. Ding et al. prepared flexible electrodes for energy harvesting devices, considering carbon black and polydimethylsiloxane rubber have low energy loss and high durability [34]. Due to the isotropic structures of the filler particles and good filler–polymer interactions, the fatigue life of the rubber composites may be very high [35]. Although the mechanical modulus at this filler level was sufficient, the fracture toughness could be improved by increasing the fracture strain value. Toughness and stretchability are two important factors for most stretchable electronic applications. Hence, the present study investigated the effects of two-dimensional MoS_2_ particles on NCB-reinforced room-temperature-vulcanized (RTV) silicone rubber composites, for tough but stretchable energy harvesting applications. The mechanical properties and fatigue under cyclic deformation were studied to determine the importance of hybrid-filler-based rubber composites. The composites were tested as electrodes in a flexible-type capacitor such as an energy harvesting device. Owing to the effects on the mechanical and piezoelectric properties of different amounts of MoS_2_, the hybrid composites can be synergized. The synergistic effects were described, followed by filler distribution and filler–polymer interactions in hybrid filler systems.

## 2. Materials and Methods

### 2.1. Materials

The silicone rubber was condensation-cured one-component room-temperature vulcanized (RTV)-silicone rubber (SR, grade KE-441) purchased from Shin-Etsu Company, Tokyo, Japan. The catalyst (CAT-RM) for room-temperature vulcanization was provided by the same company. The RTV-thinner was purchased from Shin-Etsu Company, Tokyo, Japan. Molybdenum disulfide (particle size < 2 μm) was purchased from Sigma-Aldrich. Nanocarbon black (NCB, Conductex SC Ultra grade, electrical conductivity = 2.5 S/cm [36]) was purchased from Saehan Silichem Corporation, Ltd., Seoul, Republic of Korea. All materials were used as received.

### 2.2. Characterization of Fillers

Primary characterization of the fillers was performed using an X-ray diffractometer (XpertPro-PANanalytical-Diffractometer) with an X-ray wavelength for CuKα (0.154 nm), with scanning Bragg angles (2θ) from 10° to 80°. Field emission scanning electron microscopy (FE-SEM, S-4800, Hitachi, Japan) was used to investigate the morphology of the filler structures.

### 2.3. Preparation of Rubber Nanocomposites

The different amounts of materials used for fabricating the rubber nanocomposites are provided in Table 1. The required amounts of filler(s) (in phr) were added to 100 g of RTV silicone rubber and mechanically mixed using a stirrer for 10 min. After homogeneous mixing, 2 phr vulcanizing initiator was added and thoroughly mixed for 1 min. The compounded rubber was poured into a mold and maintained for 24 h under compressed conditions. The vulcanisates were stored in a refrigerator to protect them from further curing. Before measuring the chemical and mechanical properties, the samples were removed and maintained for 24 h at room temperature (~25 °C).

### 2.4. Measurement of Cross-Link Density

The cross-link densities of the silicone rubber composites were measured using the equilibrium swelling method [37] after 7 days of immersion in toluene.

$$V_{c}=-\left[\ln\left(1-V_{r}\right)+V_{r}+\chi V_{r}^{2}\right]/V_{s}V_{r}^{1/3}$$

where *V_c_* is the cross-link density, *V_r_* is the volume fraction of rubber in the swollen sample, *χ* = 0.465 is the interaction parameter between toluene and silicone rubber [32], and *V_s_* = 106.2 is the molar volume of toluene. The *V_r_* values were calculated as follows:

$$V_{r}=\left(w_{r}/d_{r}\right)\left[\left(w_{r}/d_{r}\right)+\left(w_{s}/d_{s}\right)\right]$$

where *w_r_* is the weight of the rubber, *d_r_* is the density of the rubber, *w_s_* is the weight of the swelled toluene, and *d_s_* is the density of toluene.

### 2.5. Mechanical and Hysteresis Properties

The compressive and tensile mechanical properties were investigated using a universal testing machine (UTM, LLOYD, United Kingdom) with a 1 kN load cell. Cylindrical samples (h = 10 mm × d = 20 mm) were used for analysis of the compressive mechanical properties. For the tensile properties, dumbbell-shaped test specimens were used according to ISO 37, Type 2 (gauge length = 25 cm). The average of four tests was calculated for each reported value. For the hysteresis test, a cylindrical sample was used with 30% dynamic compressive strain over 100 cycles.

### 2.6. Filler Distribution Studies

The filler distribution was characterized using scanning electron microscopy (SEM). The level of filler dispersion was investigated, followed by an energy-dispersive spectroscopy (EDS) mapping technique fitted with the SEM instrument.

### 2.7. Fabrication of Energy Harvesting Device

Electrodes of 0.1 mm thickness were painted on both sides of the 1 mm thick unfilled elastomer slab, following the method described previously [38]. NCB (20 phr) or hybrid filler (20 phr of NCB: MoS_2_ at 17:3 ratio) was used to fabricate electrode composites, in addition to 60 phr of thinner and 100 phr of RTV silicone rubber. The painted electrodes were then coated with protective silicone rubber layers.

## 3. Results and Discussion

### 3.1. Crystal Structure and Morphology of Filler

**Figure 1:**
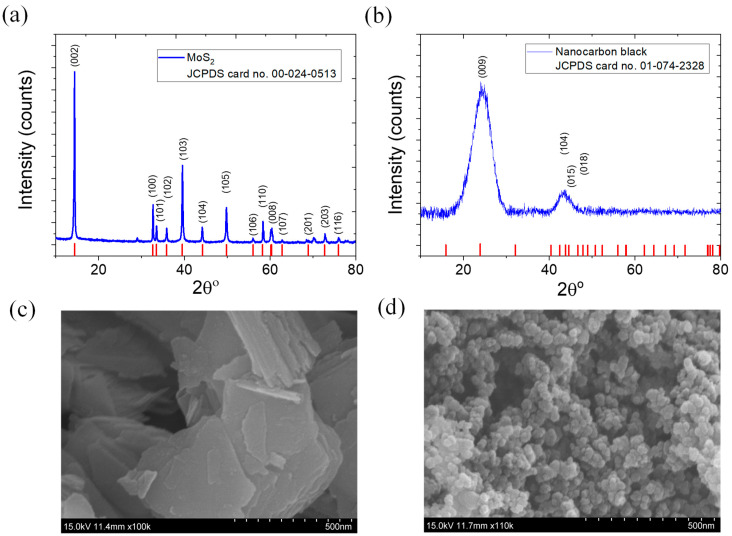
XRD plots of (**a**) MoS_2_ and (**b**) nanocarbon black; SEM images of (**c**) MoS_2_ and (**d**) nanocarbon black.

The characteristic X-ray diffraction (XRD) plots of MoS_2_ and NCB are shown in Figure 1a,b, respectively. The different major peaks in Figure 1a with 2θ values of 14.402°, 32.631°, 33.459°, 35.833°, 39.510°, 44.233°, 49.781°, 55.992°, 58.236°, 60.33°, 72.831°, and 76.009° correspond to the 002, 100, 101, 102, 103, 104, 105, 106, 110, 008, 203, and 116 crystal planes, respectively, for the hexagonal crystal system of pure molybdenum disulfide according to the reference (JCPDS card no. 00-024-0513). Similarly, the different major peaks in Figure 1b with 2θ values of 24.029°, 43.731°, 44.533°, and 47.723° correspond to the 009, 104, 015, and 018 crystal planes, respectively, of the rhombohedral crystal system of NCB, according to the card reference (JCPDS card no. 01-074-2328). Figure 1c shows an SEM image of plate-like MoS_2_ layers stacked together to form larger particles. The SEM image in Figure 1d shows spherical particles aggregated to form branched structures of NCB, which are responsible for electrical conductivity.

### 3.2. Curing Properties

**Figure 2:**
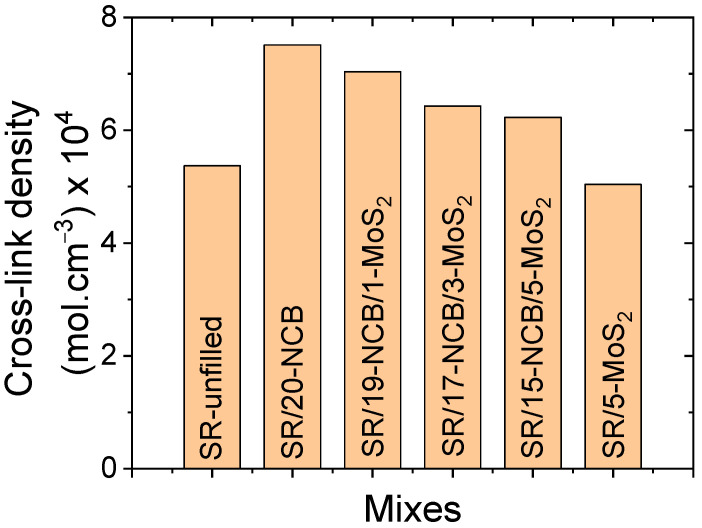
Cross-link densities of different vulcanizates.

The cross-link densities of the different rubber composites after 24 h of curing are shown in Figure 2. NCB enhanced the cross-link density, and MoS_2_ slightly reduced the cross-link density, compared to unfilled rubber. The hybrid filler-containing vulcanisates showed cross-link densities that were between the NCB and MoS_2_-only compounds. The surface chemistry of the filler particles may have been the reason for the different cross-linking densities in the different filler systems. The cross-link density in condensation-cured silicone rubber depends mainly on the curing catalyst and filler surface functionalities. Since MoS_2_ particles are platelet-shaped without hydrolyzable functional groups, they may act as a barrier to moisture, which may influence condensation-cured silicone rubber with a lower cross-link density [39,40]. In contrast, NCB promotes the cross-linking of condensation-cured silicone rubber because of its hydrolyzable surface functional groups [41].

### 3.3. Mechanical Properties of Rubber Nanocomposites

#### 3.3.1. Compressive Mechanical Properties

**Figure 3:**
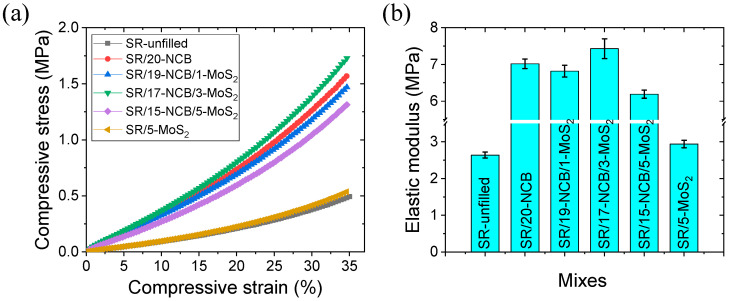
Compressive mechanical properties of different composites; (**a**) compressive stress–strain and (**b**) compressive elastic modulus.

The compressive stress–strain curves for the different rubber composites are shown in Figure 3a. From the different curves, it is evident that the compressive stress increased with slightly higher values after 15% of compressive strain. This could be due to the generation of filler percolation and polymer chain packing at higher compressive strains [42]. The addition of NCB significantly improved the compressive stress at 35% strain. Little improvement in the mechanical properties was observed with the addition of MoS_2_, unlike in the case of the unfilled rubber. This could be due to the cure retardation of sulfur-based MoS_2_ particles in silicone rubber [39,40]. The highest compressive stress at 35% of deformation was obtained for the SR/17-NCB/3-MoS_2_ hybrid composite, which was much higher than that for the unfilled rubber. The elastic modulus values of the different composites are shown in Figure 3b. The enhanced compressive stress and elastic modulus of the SR/17-NCB/3-MoS_2_ hybrid composite over those of the SR/20-NCB composite may indicate an improved filler distribution in the composite matrix [43]. The elastic modulus obtained in the compressive mode was much higher than the elastic modulus determined in the tensile mode in the present study. It is believed that the filler networks have a significant effect on the compressive modulus compared to the tensile modulus. In the compressive mode of detection, the filler particles became closer to agglomerate, which resulted in filler percolation, and improved the mechanical strength of the composite. On the other hand, filler particles became separated by the tensile strain that reduced the filler percolation, and filler networks showed slightly different behavior to the elastic modulus obtained via the tensile mode. The higher elastic modulus of SR/17-NCB/3-MoS_2_ than that of SR/20-NCB indicates more homogeneously distributed filler particles that require higher energy for packing at a higher compressive strain.

#### 3.3.2. Tensile Mechanical Properties

**Figure 4:**
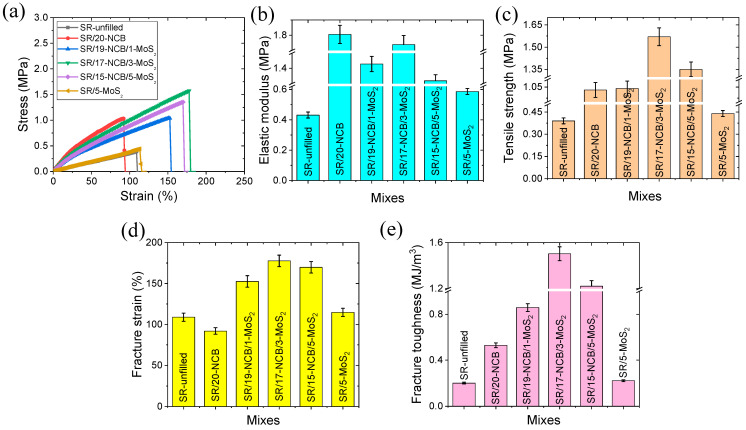
Tensile mechanical properties of different composites; (**a**) stress–strain, (**b**) elastic modulus, (**c**) tensile strength, (**d**) fracture strain, and (**e**) fracture toughness.

The different tensile mechanical properties are shown in Figure 4a–e. From the stress–strain curves in Figure 4a, it is evident that hybrid filler-containing composites provide better stretchability, and the area under the curve is also higher than those of the single and unfilled rubber composites. This could be due to the higher flexibility of the rubber matrix, followed by controlled cross-links and strong filler–polymer interactions through NCB. Thus, in the presence of MoS_2_, more flexible NCB filler networks may be formed that can withstand larger deformations. From Figure 4b of the elastic modulus chart, it is evident that the addition of MoS_2_ to the NCB-containing composites resulted in a varied elastic modulus with increasing MoS_2_ content. The SR/20-NCB composite showed the highest elastic modulus in tensile mode, which could be due to the higher reinforcing efficiency of carbon black that restricted the movement of the rubber chains. At 1 phr of MoS_2_ in the hybrid (SR/19-NCB/1-MoS_2_), the decrease in the elastic modulus was mainly due to the sum of the reduced chemical cross-links and insufficient filler dispersion. At 3 phr of MoS_2_ (SR/17-NCB/3-MoS_2_), strong and mutual dispersion [43] of fillers may have occurred, which resulted in a further increase in the elastic modulus with a subsequent increase in the tensile strength, fracture strain, and fracture toughness, as shown in Figure 4c–e. Another reason for the improved tensile strength in hybrid filler systems may be the increase in the effective surface area owing to better filler dispersion, which improves the van der Waals interactions between the polymer and filler particles [44]. Further additions of MoS_2_ may result in reduced mutual filler dispersion and a reduced number of polymer cross-links, as shown by the reduced elastic modulus in Figure 4b. Thus, MoS_2_ in the hybrid filler controls curing and improves mutual filler dispersion, which provides long-range filler–polymer connectivity. At similar modulus values in different composites, higher toughness indicates that the sample can withstand larger deformations. Hence, for stretchable electronic devices, a higher toughness is necessary to achieve higher durability. Table 2 compares the tensile properties of a few silicone rubber composites where CB is the main filler material [45,46]. From this table, it can be concluded that CB/MoS_2_ hybrid filler obtained overall good mechanical properties. Moreover, the fracture toughness was highly improved, which could be beneficial for improving the electromechanical energy harvesting, which was not reported in the literature [45,46].

#### 3.3.3. Hysteresis Losses on Dynamic Loading–Unloading Cycles

**Figure 5:**
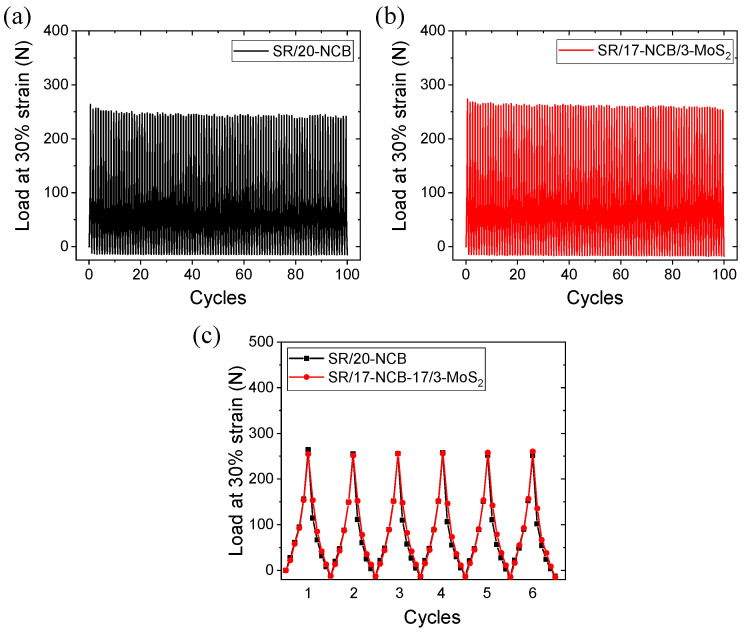
Hysteresis losses in rubber composites; (**a**) SR/20-NCB with 100 deformation cycles, (**b**) SR/17-NCB/3-MoS_2_ with 100 deformation cycles, and (**c**) SR/20-NCB & SR/17-NCB/3-MoS_2_ with first 6 deformation cycles.

The hysteresis losses in the filled compounds were mainly due to the breakdown of the filler structures and rubber networks [47]. Up to a certain deformation, the expended energy was used to break down the filler structures. After the complete breakdown of the filler structure, the expended energy caused the breakdown of the rubber networks [47]. The hysteresis loss increased up to a certain level, and then decreased because the breakdown of the filler network was proportional to the first-order deformation, while the breakdown rubber network was proportional to the second-order deformation [47]. Thus, a lower hysteresis loss signifies bonding stability. Figure 5a,b show the variations in load values of 100 cycles of cyclic compression and relaxation for up to 30% deformation of the rubber composites. From Figure 5c, it can be seen that the area under the first cyclic deformation is higher for the NCB-only compound than for the hybrid filler-loaded SR/17-NCB/3-MoS_2_ composites. In addition, Figure 5a,b show that the highest load value at maximum deformation reduced more rapidly, with increasing cycles for the SR/20-NCB composite compared to that for the hybrid filler-based SR/17-NCB/3-MoS_2_ composite. A higher number of inelastic changes, such as the permanent deformation of filler structures, indicates higher fatigue than the elastic change of filler networks [47]. In this respect, it seems that the hybrid filler containing the SR/17-NCB/3-MoS_2_ composite is more flexible, and returns to quick equilibrium networks with higher fatigue resistance than the only NCB-filled SR/20-NCB composite.

### 3.4. Filler Dispersion

**Figure 6:**
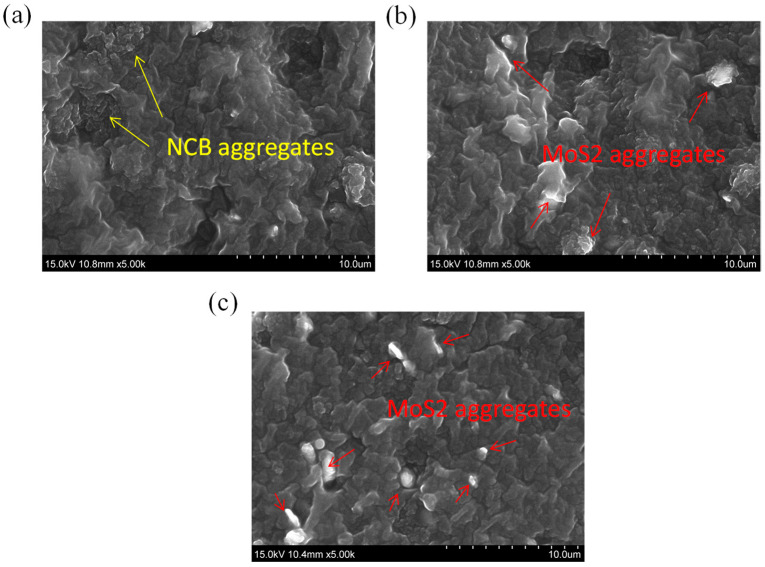
SEM images of rubber composites; (**a**) SR/20-NCB, (**b**) SR/17-NCB/3-MoS_2_, and (**c**) SR/15-NCB/5-MoS_2_.

The filler dispersion was investigated using SEM analysis. The SEM images in Figure 6a–c show that a more homogeneous filler distribution was possible in the SR/17-NCB/3-MoS_2_ composite (Figure 6b) compared with the higher MoS_2_-containing SR/15-NCB/5-MoS_2_ composite (Figure 6c), as reflected by the mechanical properties having lower values than those of the highly dispersed fillers in the SR/17-NCB/3-MoS_2_ compound. Moreover, the NCB was well dispersed in the SR/17-NCB/3-MoS_2_ composite (Figure 6b) compared to the SR/20-NCB composite (Figure 6b).

**Figure 7:**
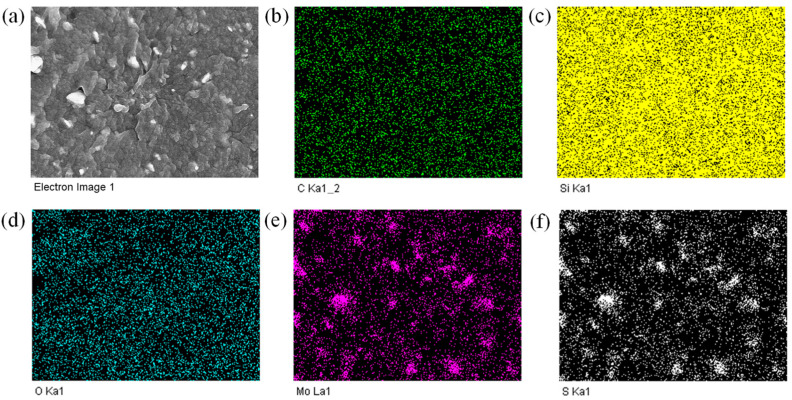
Elemental mapping of SR/15-NCB/5-MoS_2_ composite; (**a**) SEM image of mapping area, (**b**) carbon, (**c**) silicon, (**d**) oxygen, (**e**) molybdenum, and (**f**) sulfur.

The reduced filler distribution in SR/15-NCB/5-MoS_2_ can be confirmed from the EDS mapping shown in Figure 7a–f. Figure 7a shows the area under investigation. While the carbon, silicon, and oxygen elements in Figure 7b–d show homogeneity in the matrix, the MoS_2_ particles are distributed heterogeneously, as shown in Figure 7e–f. Figure 7e–f also show that nano-range filler dispersion was possible along with microdispersion. The increased nano-level filler dispersion may be due to the increased filler–filler mechanical interactions during mixing [43]. Thus, an optimum filler ratio could be the best for obtaining improved reinforcing properties.

From the mechanical properties, cross-link densities, and SEM analysis, it was evident that MoS_2_ has a small reinforcing effect on silicone rubber. However, due to its lubricating properties, it can promote the distribution of NCB particles that have greater interactions with silicone rubber. Thus, in hybrid filler systems, more homogeneously distributed fillers showed improved physical interactions with the silicone rubber matrix, and largely enhanced the fracture toughness in the hybrid composites. At 17 phr NCB and 3 phr MoS_2_, the hybrid composites showed excellent filler distribution of both NCB and MoS_2_, which is beneficial for improving the toughness value. It is believed that in highly tough rubber composites, the stress distribution is more homogenous and could result in higher electromechanical sensitivity, as found in the later section.

### 3.5. Electromechanical Sensing Performance of Energy Harvester

**Figure 8:**
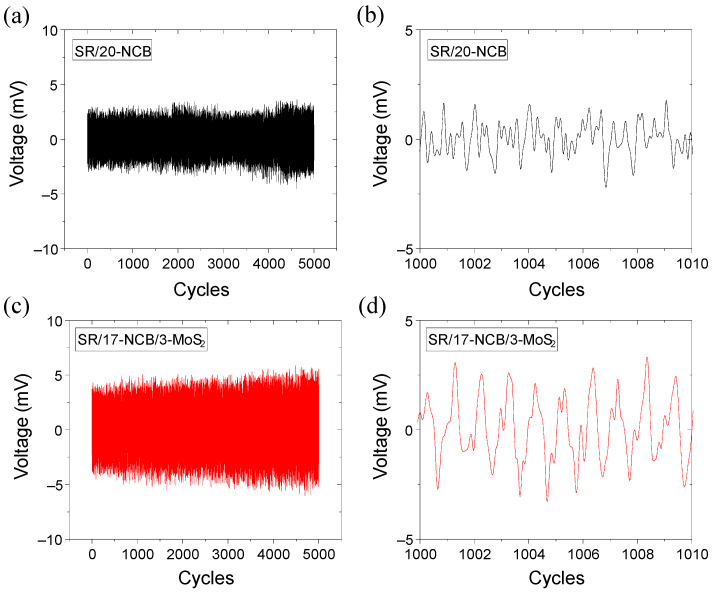
Output voltages in energy harvester with increasing cycles for selective composites as electrodes; (**a**,**b**) SR/20-NCB and (**c**,**d**) SR/17-NCB/3-MoS_2_.

To study the effect of MoS_2_ on the capacitance-based electromechanical performance, two specimens, one with 20 phr CB (SR/20-NCB) and the other with 20 phr hybrid filler containing a 17:3 CB to MoS_2_ ratio (SR/17-NCB/3-MoS_2_), were prepared according to the method described above (Section 2.7). The samples were tested for up to 5000 cycles of repeated dynamic loading and unloading in a machine, and the results are shown in Figure 8a–d. With the same applied cyclic pressure up to 2 kPa, the output voltage was much higher for the SR/17-NCB/3-MoS_2_ composite than the SR/20-NCB controlled composite. The decrease in voltage efficiency with increasing cycles may be due to the permanent breakdown of conducting NCB filler networks in the controlled composite, while a similar or increasing efficiency in the SR/17-NCB/3-MoS_2_ composite may indicate better retention of the conducting filler networks [48,49], which is also evident from Figure 5a,b, with a lower stress softening tendency for the hybrid composite. The energy output was ~100% higher in the SR/17-NCB/3-MoS_2_ composite compared to the SR/20-NCB controlled composite as an electrode. This could be due to the improved CB filler dispersion aided by MoS_2_, and the piezoresistive effect of the electronic band gap of MoS_2_ under strain [50,51] that may enhance the output voltage difference. Moreover, due to the higher toughness in the SR/17-NCB/3-MoS_2_ composite, the stress distribution was more homogeneous throughout the matrix than the SR/20-NCB controlled composite. The approximately 100% higher efficiency of SR/17-NCB/3-MoS_2_ with only 3 phr MoS_2_ compared to the control composite indicates the role of the electronic band gap of MoS_2_ in the sensitivity of the electromechanical energy harvesting performance. It was also evident from Figure 8b,d that the SR/17-NCB/3-MoS_2_ composite had very uniform sensitivity compared to the SR/20-NCB controlled composite as an electrode. Hence, two-dimensional MoS_2_ could be very useful in combination with conducting fillers for capacitance-type energy harvesters for electromechanical sensing applications [52,53,54]. Although the peak sharpness is much better in Figure 8d than in Figure 8b, this could be further enhanced by increasing the conductivity and the elasticity of the composite. Since viscoelastic materials undergo typical stress relaxation behavior and have a slow stress relaxation rate, charging and discharging are consequently not fast, and may reduce the sharpness of the peaks.

When electrical conducting filler disperses in dielectric rubber, such as in silicone rubber, a capacitor can be produced. Since the capacitance depends on the electrode surface area and the distance between electrodes, mechanical deformation can change the capacitance, followed by changing the electrode surface area and the distance between the electrodes [55]. Due to the deformation, the capacitance value of the capacitor is changed, and the charging–discharging results as negative and positive output voltages. In the hybrid filler system, it is believed that the conducting networks are distributed homogeneously in the rubber matrix, which enhances the capacitance. Since a higher capacitance belongs to a higher charge, hence the amplitudes of output voltages become higher. Moreover, in a hybrid filler system, due to the piezoelectric behavior of MoS_2_, some additional potential gradients may be generated, and can further increase the efficiency of the capacitor sensor. Such a type of capacitance-based sensing is a very low-energy process, and can be useful as a pressure sensor in health monitoring applications [55]. For example, under normal body conditions, the systolic (16 kPa) to diastolic (11 kPa) pressure difference is 5 kPa, which is much higher than the applied pressure (≤2 kPa) in this experiment. Hence, this composite can easily detect and measure blood pressure.

## 4. Conclusions

This study examines the mechanical and electromechanical sensing performance of conducting NCB and MoS_2_ hybrid fillers in RTV silicone rubber. In examining the mechanical properties and fatigue properties, it was evident that a suitable ratio of NCB and MoS_2_ in silicone rubber can provide excellent fracture toughness with improved tensile strength and fracture strain compared to unfilled and single-filler systems. Such improvements in the mechanical properties could be due to the mutually interacting fillers for excellent filler dispersion in the rubber matrix. Owing to the controlling effect of MoS_2_ on excessive cross-links in the silicone rubber matrix, the hybrid composites showed higher elongation properties, and maintained better conductivity of the NCB networks in the rubber composites. Thus, the hybrid filler with 17 phr CB and 3 phr MoS_2_ in silicone rubber provides improved mechanical properties and excellent efficiency for electromechanical sensing. A nearly 100% higher energy harvesting efficiency was obtained with ≤2 kPa of applied pressure for the 17:3 ratio of NCB to MoS_2_ hybrid compared to NCB only at 20 phr, which confirmed better electromechanical sensitivity of the hybrid composite. The improved sensitivity of the hybrid filler-loaded composite could be attributed to higher charging and discharging during capacitance change followed by the piezoelectricity of the two-dimensional MoS_2_ particles during mechanical deformation. Hence, 2D MoS_2_ could be a fascinating hybrid component filler with nanocarbon black for the future development of rubber nanocomposites with advanced mechanical and electromechanical energy harvesting and sensing applications.

## Figures and Tables

**Table 1:** Formulation table of the silicone rubber-based composites.

Formulation	RTV-SR (phr)	NCB (phr)	MoS_2_ (phr)	Vulcanizing Agent (phr)
SR-unfilled	100	-	-	2
SR/20-NCB	100	20	-	2
SR/19-NCB/1-MoS_2_	100	19	1	2
SR/17-NCB/3-MoS_2_	100	17	3	2
SR/15-NCB/5-MoS_2_	100	15	5	2
SR/5-MoS_2_	100	-	5	2

**Table 2:** Comparison of tensile properties of silicone rubber composites with carbon black as the main filler material.

Composites	Filler (Grade)	Amount of Filler	Tensile Strength (MPa)	Elongation at Break (%)	References
SR/CB (N990)	CB (N990)	20 phr	0.61	93.5	[45]
SR/CB (Vulcan XC-72)	CB (Vulcan XC-72)	20 phr	3.6	257.3	[45]
SR/CNT/CB (BP 2000)	CNT/CB (BP 2000)	5.76 vol%	4.5	211	[46]
SR/17-NCB/3-MoS_2_	CB (Conductex SC Ultra)/MoS_2_	20 phr	1.57	177.67	This study

## Data Availability

Data will be available based on request to the corresponding author.
